# Restriction beyond the restriction point: mitogen requirement for G_2 _passage

**DOI:** 10.1186/1747-1028-1-8

**Published:** 2006-05-18

**Authors:** Floris Foijer, Hein te Riele

**Affiliations:** 1 Division of Molecular Biology, the Netherlands Cancer Institute, Plesmanlaan 121, 1066CX, Amsterdam, The Netherlands

## Abstract

Cell proliferation is dependent on mitogenic signalling. When absent, normal cells cannot pass the G_1 _restriction point, resulting in cell cycle arrest. Passage through the G_1 _restriction point involves inactivation of the retinoblastoma protein family. Consequently, loss of the retinoblastoma protein family leads to loss of the G_1 _restriction point. Recent work in our lab has revealed that cells possess yet another mechanism that restricts proliferation in the absence of mitogens: arrest in the G_2 _phase of the cell cycle. Here, we discuss the similarities and differences between these restriction points and the roles of cyclin-dependent kinase inhibitors (CKIs) herein.

## Introduction

During each division cycle, cells need to duplicate their genome and distribute the two copies equally over the two daughter cells. The processes of DNA-duplication (S-phase) and cell division (mitosis) are separated by two gap phases, G_1 _and G_2_, respectively. During these phases, several mechanisms operate to prevent cells from continuing the cell cycle under inappropriate conditions such as the absence of growth factors or the presence of DNA damage. The gap phases provide a window of time during which cells assess whether the environment still favours proliferation (during G_1_) or whether S-phase was performed correctly (during G_2_). If this is not the case, normal cells can interrupt the cell cycle in the gap phases through growth inhibitory mechanisms that activate the retinoblastoma proteins or the p53 transcription factor. In cancer cells, these growth inhibitory pathways are often disrupted, leading to unscheduled proliferation[1].

## The G_1 _restriction point

One critical environmental factor for cell proliferation is the presence of growth factors and normal cells respond to their absence with cell cycle arrest in G_1_. However, during the G_1 _phase, growth factors are only required until 2–3 hours prior to initiation of S-phase[2]. This moment in G_1 _was first described in 1974 by Arthur Pardee and termed the restriction point R. Pardee found that cells that have passed the G_1 _restriction point can progress through S-phase and complete mitosis independently of mitogens[3]. Since entry into S-phase after growth factor induction was found to rely on protein synthesis, it was suggested that cells need to accumulate a protein in order to pass the restriction point[4]. This hypothetical protein was referred to as the R-protein, and is apparently induced by mitogens. Importantly, Pardee found that the restriction point was defective in cancer cell lines, providing physiological relevance for the restriction point. In addition, cancer cells were much more resistant to inhibition of protein synthesis, suggesting that the R-protein was either stabilized in these cells or not required[5]. The transformed cell lines that were used in this study carried simian virus 40 (SV40)[2]. The finding that the oncogenic products of DNA tumor viruses, such as SV40 large T antigen, adenovirus E1A and HPV E7, disrupt G_1_/S control through their inhibitory interaction with the retinoblastoma gene product[6,7], provided a crucial link to the machinery underlying the restriction point.

The retinoblastoma gene encodes a 105 kD nuclear phosphoprotein (pRB) that in its unphosphorylated state can bind to and repress E2F transcription factors whose activity is essential for G_1_/S transition [8-12]. Since pRB is dephosphorylated late in mitosis by PP1 phosphatase[13], it needs to be phosphorylated during G_1 _to allow entry into S-phase and this requires mitogenic signalling. Mitogenic signalling results in increased transcription and stabilization of CYCLIN D [14], which stimulates its catalytic partners CDK4 and CDK6 to phosphorylate pRB early in G_1_, causing partial inactivation of pRB and release of E2F[15]. E2F transcription factor activity results in increased transcription of several genes involved in cell cycle progression among which *CYCLIN E*. CYCLIN E/CDK2 activity phosphorylates pRB to a higher extent, triggering full release of E2F and onset of S-phase. Conversely, in the absence of mitogens, decreased transcription of *CYCLIN D1 *and decreased stability of CYCLIN D1 protein favor the pRB unphosphorylated state, which inhibits E2F activity and causes cell cycle arrest in G_1_. Additionally, mitogen deprivation causes accumulation of the cyclin dependent kinase inhibitor (CKI) p27^KIP1 ^through activation of the FOXO transcription factor[16,17]. p27^KIP1 ^is a potent inhibitor of CYCLIN E/CDK2 kinase activity[18], and will therefore prevent inactivation of pRB.

Somewhat unexpectedly, *Rb*-deficient mouse embryonic fibroblasts (MEFs) still arrested in G_1 _when mitogen starved, although a small fraction of the cells could enter S-phase[19,20]. This has been explained by the activity of two other retinoblastoma protein family members, p130 and p107, which have redundant functions in regulating E2F transcription factors[21]. Together, these proteins make up the so-called family of pocket proteins, which refers to their highly conserved 'pocket-region' that is essential for interacting with E2Fs[10,22,23]. Indeed, MEFs that have lost all three pocket proteins are no longer capable of arresting in G_1 _when mitogen starved[24,25].

The retinoblastoma proteins can thus be seen as molecular switches that operate at the restriction point: when switched -*off*- by mitogens, they allow passage through the restriction point and initiation of S-phase, while the -*on*- state results in cell cycle arrest. The downstream target of the switches are the E2F transcription factors, whose activity results S-phase entry[12]. The switches are operated by cyclin-associated kinase activities in G_1 _that can be modulated by the stability of the cyclin subunit, as is the case for CYCLIN D, or by inhibition of the kinase activity, as is the case for CYCLIN E/CDK2. CYCLIN D has been suggested as an appropriate candidate for the R-protein[26], since it is dependent on mitogens for its synthesis, is destabilized in the absence of mitogens and operates the 'molecular switch'. However, ablation of all three CYCLIN D family members (*Cyclin D1, D2 and D3*) did not block re-stimulation of serum-arrested cells (*i.e*., 60–80% of the cells were able to re-enter the cell cycle when stimulated with 10% serum)[27]. In contrast, MEFs in which both CYCLIN E family members (CYCLIN E1 and E2) were ablated, failed to re-enter the cell cycle after mitogen deprivation due to failure in loading MCM proteins to the DNA, which is essential for S-phase initiation[28,29]. Since CYCLIN E accumulates during G_1 _and its ablation results in failure of cell cycle re-entry, CYCLIN E may be a good candidate for the R-protein[30].

## Mitogen dependence of *Rb/p107/p130*-deficient MEFs

Pardee originally suggested that once cells have passed the restriction point, the cell cycle can proceed independently of mitogens until mitosis[2]. Accordingly, ablation of the retinoblastoma gene family, resulting in complete loss of the G_1 _restriction point[24,25], should allow mitogen-independent proliferation. However, this was shown not to be the case: pocket-protein deficient cells are prevented from entering mitosis in the absence of mitogens by two mechanisms: (*1*) the majority of cells undergoes apoptosis[24,25,31]; (*2*) surviving cells arrest in the G_2 _phase of the cell cycle within 3–5 days[31]. Apparently, mitogenic signaling is not only required for passing the G_1 _restriction point, but also for passage through G_2_. While activation of the G_1 _restriction point in normal cells involves inhibition of D- and E-type cyclins, mitogen-starvation-induced G_2 _arrest is effected by accumulation of p27^KIP1 ^and p21^CIP1 ^that act as inhibitors of CYCLIN B1- and CYCLIN A-associated kinase activities[31].

CKI mediated inhibition of CDK1, the catalytic partner of CYCLIN B1, has been described in other systems as well. In addition to its CDK2-inhibiting activity[32], p21^CIP1 ^was shown to induce a G_2 _arrest upon DNA damage[33] or upon over-expression[34] by inhibiting CDK1 kinase activity through direct interaction. In contrast to an earlier report[18], recent work from several laboratories has revealed that also p27^KIP1 ^can inhibit CDK1 kinase activity through direct interaction. *E.g*., p27^KIP1 ^is highly expressed in thymocytes and splenocytes and binds to and inhibits CYCLIN B1-CDK1 kinase activity in these cells[35]. In mice, ablation of SKP2, an F-Box protein that targets p27^KIP1 ^to an SCF ubiquitin-ligase complex, resulted in elevated p27^KIP1 ^levels associated to CDK1. Most defects in these animals are the result of decreased CDK1 and CDK2 kinase activities and can be rescued by concomitant ablation of p27^KIP1^, which restores physiological cyclin-dependent kinase activities[36].

## G_2 _arrest: a second restriction point?

**Figure 1:**
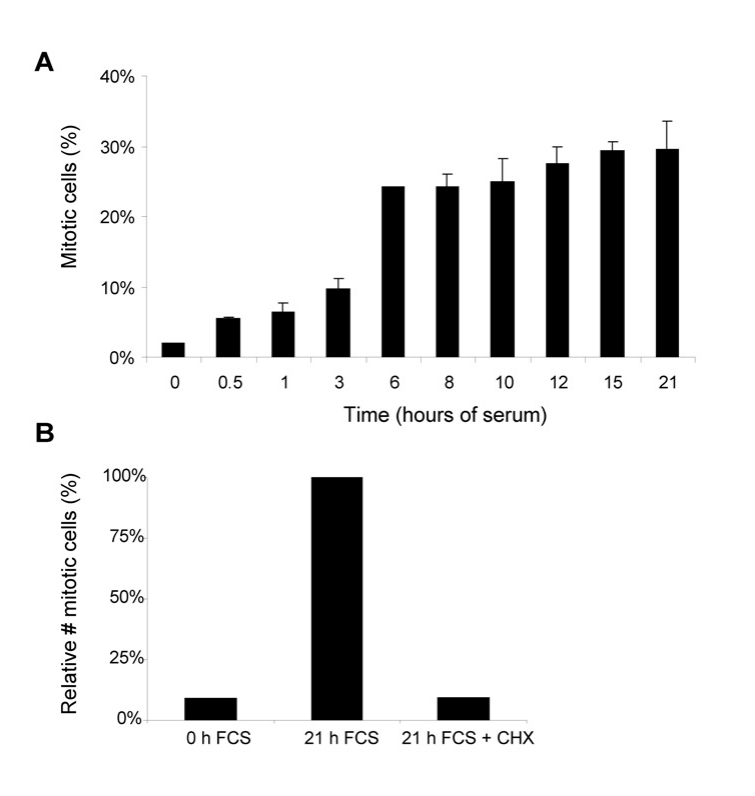
**Evidence for a G_2 _restriction point. A. **Cell cycle re-entry from G_2 _requires 6 hours of mitogen-stimulation. Serum-starved cells were stimulated by the addition of serum-containing medium. Subsequently, at the indicated times medium was replaced with serum-free medium containing Taxol for the last 9 hours. At 21 hours cells were harvested and fixed in 70% ethanol and mitotic entry was determined by MPM2 FACS staining. Error bars indicate the standard deviation for two experiments. **B. **Cell cycle re-entry from G_2 _requires protein synthesis. Serum-starved cells were serum-stimulated in the absence or presence of 50 μg/ml cycloheximide (CHX). Cells were fixed at 21 hours and mitotic entry was determined by MPM2 FACS staining. The level of MPM2 positivity in serum-stimulated cells at 21 hours is set at 100%.

The mitogen-starvation-induced G_2 _arrest shows several similarities to the G_1 _restriction point. *E.g*., both depend on inhibition of cyclin-associated kinase activities and in both, accumulation of p27^KIP1 ^plays an important (although not exclusive) role[31]. Importantly, both are reversible: mitogen stimulation of G_2_-arrested pocket-protein-deficient cells results in reactivation of the cell cycle and synchronous entry into mitosis after approximately 15 hours. Is there also a true restriction point in G_2 _in the sense that a time point can be identified after which cells do no longer require serum to enter mitosis? To address this issue, we serum-starved pocket-protein deficient MEFs for 7 days, and then re-fed the cells with serum-containing medium at time point 0. At several time points hereafter, we replaced the serum-containing medium for serum-free medium. To quantify G_2 _exit, we trapped the cells in mitosis using the microtubule-stabilizing drug Taxol. 21 hours after serum-stimulation, we harvested the cells and determined the mitotic fraction by FACS-staining for the mitotic marker MPM2 as described previously[31]. Figure 1A shows that the fraction of cells entering mitosis gradually increased upon longer duration of serum exposure. However, at 6 hours of serum exposure, the maximum amount of mitotic cells was reached. This indicates that mitogen-starved G_2 _arrested cells only required a window of 3–6 hours of serum in order to re-enter the cell cycle, identifying a G_2 _restriction point at approximately 10 hours before mitotic entry.

Next, we wondered whether cell cycle re-entry of serum-starved G_2_-arrested cells relies on protein translation, as was previously shown for recovery from G_1 _arrest. We therefore compared serum stimulation of G_2_-arrested cells in the presence and absence of the translation inhibitor cycloheximide. Figure 1B shows that inhibition of protein synthesis precluded cell cycle re-entry of serum-stimulated cells. This suggests that passage through the G_2 _restriction, like passage through the G_1 _restriction point, depends on synthesis of one or multiple proteins.

An important question now is: why was the G_2 _restriction point not identified in the original experiments of Pardee? A first explanation is that activation of pocket proteins in serum-starved normal cells (*i.e*., wild type MEFs) imposes an arrest in G_1 _that largely prevents subsequent cell cycle events. However, if cells possess two restriction points, and mitogen deprivation results in inhibition of all cyclin-associated kinase activities, why then do normal cells mainly arrest in G_1 _and is G_2 _arrest only seen in pocket-protein compromised MEFs? One reason could be that the levels of suppression of CYCLIN/CDK activity required for G_1 _or G_2 _arrest are different. In wild type cells, minor inhibition of D- and E-type cyclins may already impose a G_1 _arrest through accumulation of hypophosphorylated pocket proteins. In contrast, G_2 _arrest imposed by inhibition of CYCLIN A- and B kinase activities requires high levels of p21^CIP1 ^and p27^KIP1^, which need several days to accumulate. Apparently, when these levels are reached in pocket-protein-deficient cells, the remaining CDK2 kinase activity is still sufficient to drive cells through S-phase, while the remaining CDK1 activity is too low to allow entry into mitosis, resulting in G_2 _arrest.

Secondly, G_2 _arrest in serum-starved, pocket-protein defective cells relies on functional p53[31]. The cancer cell line that was used for the original experiments contained SV40 Large T antigen, which inactivates the pocket proteins, but also p53[37]. Therefore, both the G_1 _and the G_2 _restriction points were inactivated in these cells.

## Conclusion

The G_1 _restriction point defines a window of mitogen requirement in G_1_. However, in the absence of pocket protein activity, another growth-restricting mechanism in G_2 _becomes manifest that prevents unconstrained proliferation under mitogen-starved conditions. This G_2 _arrest has the following features:

1. It allows cell cycle progression only in the presence of mitogens.

2. It is reversible: mitogen-starved, G_2_-arrested cells re-enter the cell cycle synchronously upon mitogen stimulation.

3. A specific moment in G_2 _exists, approximately 10 hours before mitotic entry, after which cells can progress into mitosis independently of mitogens.

4. Recovery from G_2 _arrest relies on accumulation of one or multiple proteins.

5. The G_2 _arrest is effectuated by inhibition of CYCLIN-CDK activity through association with CKIs.

These properties of serum-starvation induced G_2 _arrest identify a true restriction point in G_2_. However, the G_1 _and G_2 _restriction points are not completely identical at the molecular level. For one: whereas the G_1 _restriction point critically depends on the activity of the pocket proteins, the G_2 _restriction point only becomes manifest when pocket protein activity is diminished or absent. Furthermore, the G_1 _restriction point involves degradation of CYCLIN D in addition to CKI-mediated inhibition of CYCLIN E, whereas the G_2 _restriction point appears to rely solely on CKI-mediated inhibition of CYCLIN A- and CYCLIN B- associated kinase activities.

**Figure 2:**
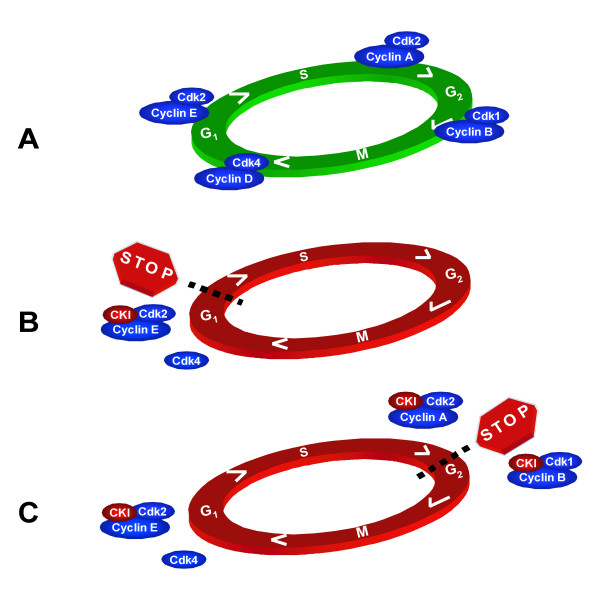
**Extending the restriction point. A. **Cell cycle progression is dependent on CYCLIN-CDK kinase activity. **B. **Mitogen starvation results in cell cycle arrest in G_1 _through degradation or suppression of CYCLIN D and CKI-mediated inhibition of CYCLIN E-CDK2. **C. **Unscheduled passage through the G_1 _restriction point in the absence of mitogens (*e.g*., through RB loss) results in cell cycle arrest in G_2_.

Taken together, we postulate that cells possess *two *restriction points defining the requirement for mitogenic signaling in G_1 _*and *in G_2 _to stimulate CYCLIN D/E and CYCLIN A/B kinase activities, respectively (Fig. 2A). In both, accumulation of p27^KIP1 ^plays an important role. When growth factors are removed from normal cells, rapid disappearance of CYCLIN D1 and inhibition of CYCLIN E by accumulation of p27^KIP1 ^results in hypophosphorylated pRB, low E2F activity and G_1 _arrest (Fig. 2B). In cells that have lost the pocket proteins and hence the G_1 _restriction point, the G_2 _restriction point comes into play. Accumulation of p21^CIP1 ^and p27^KIP1 ^apparently leaves sufficient CDK2 activity to allow cells to cross the G_1_/S border and complete S phase (likely because of elevated E2F activity in the absence of pocket proteins). However, inhibition of CYCLIN A- and B kinase activity now arrests cells in G_2 _(Fig. 2C).

We envisage that the G_2 _restriction point serves as a backup mechanism to prevent unconstrained proliferation of cells that have lost proper G_1_/S control. Indeed, a substantial amount of circumstantial evidence suggests a role for the G_2 _restriction point in the suppression of cancer[38]. *E.g*., it is possible that tumor cells in a primary tumor retain a normal G_2 _arrest that does not perturb proliferation at the site of origin but only becomes activated under special conditions such as dissemination to distant sites. Indeed, occult, non-proliferating tumor cells that were found in the bone marrow and bloodstream of cancer patients without overt metastases, may present an example of this scenario[39]. Elucidation of the mechanism of cell cycle arrest is of paramount importance to control the behavior of such cells.

## Abbreviations

MEFs: mouse embryonic fibroblasts

CKI: cyclin dependent kinase inhibitor

FCS: fetal calf serum.

## Competing interests

The author(s) declare that they have no competing interests.
